# Supplementary material for: Network intrusion detection using a hybrid graph-based convolutional network and transformer architecture
Source: PLoS One. 2026 Jan 21;21(1):e0340997. doi: 10.1371/journal.pone.0340997 (PMC12822977; doi:10.1371/journal.pone.0340997)
Supplement: Attachment — Submitted filename: Response to Reviewers.docx [file pone.0340997.s002.docx]

**Dear Emily Chenette,**

Editor-in-Chief

**RESPONSE TO REVIEWERS – REVISED MANUSCRIPT SUBMISSION**

**Manuscript Title:** Network Intrusion Detection Using a Hybrid Graph-based Convolutional Network and Transformer Architecture

**Manuscript ID:** PONE-D-25-45283

We are grateful for the opportunity to revise and resubmit our manuscript titled “Network Intrusion Detection Using a Hybrid Graph-based Convolutional Network and Transformer Architecture”. We sincerely appreciate the insightful and constructive comments provided by the reviewers and the academic editor. Their feedback has significantly improved the clarity, quality, and overall contribution of our work.

We have carefully addressed each comment in detail and revised the manuscript accordingly. Below is a comprehensive table summarizing the reviewers’ comments alongside our corresponding responses. All changes made to the manuscript have been clearly incorporated in the revised version, and page/section numbers have been indicated where appropriate.

**DEFICIENCY TABLE**

| **Reviewer’s Comment** | **Author’s Response / Comment** |
| --- | --- |
|  |  |
| ***Reviewer #1 Comments*** |  |
| 1. Consistency in results: Ensure that performance metrics (training/validation/test accuracies) are consistently reported across Abstract, Results, and Conclusion. | We have reviewed the manuscript to ensure consistent reporting of training, validation, and test performance metrics across the Abstract, Results, and Conclusion sections. All numerical values have been aligned to maintain clarity and accuracy. |
| 2. Broader dataset validation: Since only one dataset (CIC-IDS 2018, Feb 14 subset) was used, the generalizability claim is limited. PLOS ONE usually requires broader evidence or at least a stronger discussion of limitations. | We have added a discussion on the limitation of using only the CIC-IDS 2018 dataset and emphasized that results may vary with other datasets. We also suggested future work involving multiple datasets to validate generalizability. |
| 3. Data availability: Already compliant (dataset is public), but consider including the exact preprocessing code repository for transparency. | We have provided a link to the preprocessing and model implementation code repository in the Data section to ensure transparency and reproducibility. |
| 4. Writing quality: Some grammatical issues and repetitive phrasing in Introduction/Related Works could be polished for readability. | We have revised the Introduction and Related Works sections to correct grammatical errors and reduce repetitive phrasing, improving overall readability. |
| 5. Impact framing: Strengthen the claim of practical significance (e.g., efficiency in real-world cloud/IoT deployments, lower resource requirements compared to deep transformers). | We have revised the Discussion and Conclusion sections to highlight the practical significance of our approach, emphasizing its efficiency, lower computational and memory requirements, and suitability for deployment in real-world cloud and IoT environments compared to larger transformer-based models. |
|  |  |
|  |  |
| ***Reviewer #2 Comments*** | **Author’s Response / Comm** |
| 1. Grouping rows by time window and class is followed by connecting each node to its 50 closest neighbors. The input graph is constructed using only time window information and k-NN in feature space, without utilizing true labels. | This is partially addressed in the notebook, with modifications for flexibility and to avoid label leakage. The graph construction uses time-window grouping but does not group by class/label to prevent any use of true labels in graph building. Instead, rows (flows) are grouped solely by time windows, and edges are formed via k-NN in the feature space (15 selected features after preprocessing, standardized using StandardScaler). |
| 2. Avoid performing random shuffles. Conduct chronological (or day-wise) partitioning on the CIC-IDS2018 dataset (for example, training on earlier days and testing on a later day). Generate distinct graphs for each partition (ensuring no edges cross between splits), or employ mini-batches that conceal message passing to prevent the GNN from observing validation/test nodes during training. Provide results as the mean ±95% confidence interval using a minimum of 5 different seeds. | The dataset is loaded and strictly sorted by date, with no random shuffling, and then partitioned chronologically so that earlier dates form the training set, the next date the validation set, and the final date the test set (e.g., Train: ['02-16-2018', '02-28-2018'], Val: ['03-01-2018'], Test: ['03-02-2018'], corresponding to roughly 60.5%, 11.3%, and 28.2% of the data). Graph construction is performed in a split-aware manner using train/val/test masks to ensure that no edges connect nodes across partitions, which is confirmed by outputs indicating zero cross-split edges. To ensure reproducibility, the entire graph-generation process is repeated under five different random seeds (23, 42, 123, 456, 789), each controlling sampling and k-NN behavior. |
| 3. Provide the precise measurement method (Euclidean or Cosine), feature standardization utilized in k-NN, the examined values of k, clarity on directed or undirected edges, and methodology for adjacency normalization. Include experimental variations: k in {10, 25, 50, 100}, with or without time-window restriction. | Both cosine and Euclidean distances are used when constructing k-NN graphs, with the loop iterating over metrics ['cosine', 'euclidean'], and all features are standardized using scikit-learn’s StandardScaler after selecting the 15 numerical inputs and cleaning infinities/NaNs. The exact neighborhood sizes examined are {10, 25, 50, 100}, and although edges originate as directed from the k-NN process, they are finalized as undirected in the stored graphs. Adjacency normalization is not applied during data preparation but is intended for model training via symmetric normalization in PyTorch Geometric’s GCN layers. A full factorial set of variations is run time-window vs. no time-window, all k values, and both metrics yielding 80 total graphs across 5 seeds. |
| 4. Compare the performance of models using GCN-only, Transformer-only, MLP, and a strong tabular baseline (e.g., XGBoost) on the same dataset split. Additionally, conduct component ablations by removing the GCN and Transformer components to assess the added value of the hybrid model over simpler ones. This analysis validates that the hybrid model offers tangible benefits beyond basic models. | This stage is not yet completed due to compute limits, but the plan is to train a set of baselines and ablations including a 2-layer GCN, a Transformer-only model, an MLP, an XGBoost tabular baseline, the hybrid GCN Transformer model, and ablated hybrids that remove either the GCN or Transformer component, using the previously generated graphs and chronological splits, training on the train mask, tuning on validation, and evaluating on test under the same five seeds for mean ± 95% CI. |
| 5. Provide detailed performance metrics including per-class precision, recall, F1 score (macro and weighted), PR-AUC, ROC-AUC, confusion matrices, and calibration (ECE) on the test set. Present the test accuracy as a single value consistent throughout the report. | This evaluation stage is still pending training but will report a consistent set of test-only metrics, including per-class precision/recall/F1, macro and weighted F1, PR-AUC, ROC-AUC (one-vs-rest), a confusion matrix, Expected Calibration Error, and a single headline test accuracy. These will be computed with sklearn.metrics and torchmetrics, visualized as tables and figures, and kept consistent across the report. The evaluation step is lightweight and will run in under five minutes per model once training from Point 4 is complete. |
| 6. Provide a repository containing graph-building scripts, split masks, configurations, code, and random seeds. Include a list of package versions and hardware specifications. Clearly document hyperparameter search details, specifying the ranges, patience, and conditions for early stopping. Add descriptive captions to figures and diagrams that accurately reflect the pipeline implementation. | We have created a public repository that includes all graph-building scripts, split masks, configuration files, source code, and random seeds. The repository also provides a complete list of package versions and hardware specifications used in our experiments. Additionally, we have documented the hyperparameter search process, including parameter ranges, patience values, and early-stopping conditions. Figure and diagram captions have been updated to provide clear descriptions that accurately reflect the implemented pipeline. |

We believe that the revisions we have made substantially strengthen the manuscript, and we hope the updated version meets the expectations of the reviewers and the academic editor. We appreciate your time and consideration, and we look forward to your favorable response.

Thank you once again.

Sincerely,

Emmanuel Botchway
